# Predictive Factors of Anxiety, Depression, and Health-Related Quality of Life in Community-Dwelling and Institutionalized Elderly during the COVID-19 Pandemic

**DOI:** 10.3390/ijerph191710913

**Published:** 2022-09-01

**Authors:** Stefania Pascut, Susanna Feruglio, Cristiano Crescentini, Alessio Matiz

**Affiliations:** 1 Department of Languages and Literatures, Communication, Education and Society, University of Udine, 33100 Udine, Italy; 2 Department of Psychology, Sapienza University of Rome, 00185 Rome, Italy; 3 WHO Healthy Cities Project, Municipality of Udine, 33100 Udine, Italy; 4 Institute of Mechanical Intelligence, Scuola Superiore Sant’Anna, 56127 Pisa, Italy

**Keywords:** COVID-19 pandemic, health-related quality of life, older people, loneliness, anxiety, depression, spirituality, community-dwelling elderly, institutionalized elderly

## Abstract

The COVID-19 health emergency and restrictive measures have increased psychological problems, particularly anxiety and depression, in the general population. However, little is known about mental health conditions and the possible risk and protective factors of specific population groups, such as institutionalized vs. community-dwelling elderly. We investigated the abovementioned aspects in a sample of 65–89-year-old people during the third wave of COVID-19 in Italy. We employed a sociodemographic survey and four questionnaires on health-related quality of life (SF-36), loneliness (UCLA), spirituality (FACIT-Sp), and anxiety/depression (HADS). Our findings suggest that the physical, psychological, and spiritual well-being of the elderly had not been seriously impaired by the events related to the pandemic, although most of the participants reported a worsening of their social life and a moderate/high fear of COVID-19. In regression analyses, these two latter aspects turned out to be predictors of higher anxiety, while spiritual well-being and the possibility to get out of the house/institution emerged as protective factors against anxiety and for preserving quality of life, respectively. Our findings help refine the picture of the condition of the elderly in the aftermath of the pandemic, giving some hints about how to continue supporting their well-being and quality of life.

## 1. Introduction

Since December 2019, the world has been experiencing the challenge of a new coronavirus disease (SARS-CoV-2). Several preventive measures have been taken by governments, such as quarantine, physical distancing, and travel restrictions that, nevertheless, appeared to have impacted on the psychophysical well-being of individuals, increasing their risk of mental health problems. It is indeed known from the previous literature that the reduction of social contact, the excess of information, and the apprehension about the spreading of the virus, could all be factors of depression, anxiety, and emotional instability, also acting as amplifying factors of pre-existing clinical conditions [1,2,3,4]. It is estimated that between one-third and one-half of the population will develop or increase psychopathological disorders during the pandemic period if preventive measures are not taken to reduce its possible negative effects [5]. However, to date, much of the literature has focused on the immediate effects of the first pandemic wave, while the effects of the subsequent waves have been much less studied; thus, it is not clear how individuals are responding and adapting to the long-lasting emergency situation. Moreover, large inter-individual differences exist, and for future pandemics, there is a clear need to comprehensively assess individuals’ resilience from the start to provide personalized help and interventions tailored to the specific needs of, in particular, vulnerable groups [6].

Although the virus can affect people of any age, older people appeared as the most vulnerable population group and the most severely affected [7]. In an attempt to redefine vulnerability in the era of COVID-19, a recent study described the elderly as a group “disproportionally exposed to risk” [8], not only because of the direct contact with the virus, but also because of indirect effects of social isolation, loneliness, and lack of access to healthcare resources [9,10], deriving from the difficulty to cultivate social relationships and be part of the community life.

Considering the importance of reflecting on the critical points and lesson learnt from the COVID-19 pandemic to develop effective strategies and actions and increase preparedness to other future emergency situations, we decided to explore the impact of the stressful COVID-19 pandemic on older people’s mental health condition. We focused particularly on this population group since the previous literature had mainly addressed the effects of COVID-19 pandemic on young and middle-aged people [11,12,13,14,15,16]. However, populations are growing older all over the world, and this demographic trend will have a great impact in terms of needs to be answered to, services to be reorganized, and resilience to be promoted; thus, further research is needed on this population target.

More specifically, we wanted to investigate, in an elderly population, the possible changes in the conditions of health-related quality of life, anxiety, and depression as a consequence of the stress experienced during the pandemic. As it is known that older people had experienced conditions such as sickness, loneliness, anxiety, panic, stigma, and death anxiety in previous epidemic periods [17], it is likely that similar problems could be experienced also during the COVID-19 pandemic [18,19,20]. However, the prevalence of anxiety and depression in the elderly is controversial. In some studies, anxiety and depression increased particularly due to reasons such as physical problems, movement limitations, and dependency on others [21,22,23]. Some studies, on the contrary, showed lower levels of anxiety and depression, and they motivated this result with a possible higher resilience and coping mechanisms in older people in comparison to the younger population or with a greater acceptance of death with maturity developed by age [24,25]. Moreover, when one considers resilience in the context of the COVID-19 pandemic, a few studies have shown that older people seem to better manage their emotional and psychological consequences [26,27,28,29].

Alongside the impact of the COVID-19 pandemic on older people’s physical and mental health, we intended to investigate the role and possible influence of loneliness, healthy lifestyles, and spirituality.

Loneliness, together with social isolation, has a great impact on well-being [30,31,32,33] and may result in a lowering of mood and cognitive stimuli, altering the regulation of inflammatory responses in the body, thus damaging the immune system, the ability to concentrate, and sleep habits [34,35,36]. In older age, social isolation and loneliness increase the risks of cardiovascular disease, stroke, diabetes, cognitive decline, dementia, depression, anxiety, and suicide [37,38,39,40]. However, they are still largely neglected social determinants of health and public health concerns in the elderly, although the COVID-19 pandemic and the physical-distancing measures have increased the salience of these issues [8,41,42,43,44]. As an example, in the general population, a recent study involving 1006 Italians during the first COVID-19 lockdown showed that a longer isolation correlated with a worse mental health status (e.g., depression) [45]. However, many questions and uncertainties remain to be addressed by the research community since still little evidence is available on this topic and effective interventions are needed [46,47].

Another important aspect which could play a role as a protective factor during the pandemic was the possibility of maintaining healthy lifestyles, intended primarily as continuing engaging in physical and recreational activities of different kinds (i.e., leisure moments, music, painting, reading, playing, etc.). We know from the previous literature, carried out both within the current health emergency [48,49,50] and before it began [51,52], that a physically and intellectually active lifestyle has a positive impact against internalizing problems, especially in favor of depressive symptoms. For example, in a sample of healthy adults, Crescentini et al. [14] suggested the importance of not giving up physical activity even during periods of isolation and social confinement, possibly underlining its importance through targeted support interventions. Other studies confirmed that changes in lifestyle factors, including nutrition, exercise, smoking, alcohol consumption, screen time, and sleep, may be able not only to contribute to shifting the risk distribution for COVID-19 [53], but also appear to play a role in the management of mental disorders [54], which are commonly observed in pandemics such as the current one [55,56]. However, considering that most of the previous literature addresses young and middle-aged people, we wanted to explore if our sample corroborated these findings.

Spiritual support may also be a strategy for individuals to cope with life stressors, helping in the search for meaning and in overcoming loneliness and coping with reality. Spirituality is one of the most valid tools for the elderly to interpret and make sense of what has happened during the pandemic [57], as well as to cope with difficulties and overcome loneliness, stress, depression, death anxiety, and similar problems [58,59,60], besides being an essential part in certain medical fields such as the palliative care [61]. Faith and spirituality are not strictly connected with religious beliefs but could also be expressed by spending time in meditation, listening to inspirational programs, reading uplifting literature, and caring for others in need [62], especially in the sense of staying active in the community by delivering care/help for frail people [63]. For these reasons, we also aimed at exploring if spirituality had a protective role against the effects of the COVID-19 pandemic on older people.

Finally, among the elderly, those institutionalized are worth higher attention, due to the risk of loneliness and social isolation, deriving from the closure of healthcare facilities and the ban of visits by parents, relatives, and friends. For this reason, institutionalization could have contributed to worsening older people’s mental health problems, such as internalizing symptoms, and their physical and psychological well-being [64,65,66]. Thus, in the present study, we also compared the living conditions of community-dwelling versus institutionalized older people.

To sum up, the aim of this study was to investigate the status of physical and mental health of a sample of older people during the third wave of COVID-19 in Italy. This might be of special interest also due to the fact that Italy was the first country in Europe to implement a nationwide lockdown and to introduce the most stringent restrictive measures to contain the spreading of the virus. We assessed older people’s health-related quality of life and levels of anxiety and depression, and we explored as possible predictors their levels of loneliness, healthy lifestyles, and spirituality. A structured survey was designed and administered to both home-dwelling and institutionalized elderly people to provide a comprehensive picture of the effects of the health emergency and restrictive measures in this age group (65–89 years). Conducting research on these issues could be useful to better understand older people’s perceived care needs and psycho-emotional concerns and to invest in interventions and strategies aimed at responding to these needs [67,68]. A better understanding of the complex interactions of cognitive, emotional, physical, and social aspects of older people’s mental health will also help to offer more effective services and develop intervention programs for the elderly [3].

## 2. Materials and Methods

### 2.1. Overview of Procedure

This study involved a sample of older people living in the community and in healthcare facilities. As the data were collected in 2021 and referred to a period (April–July 2021) of variable restrictive measures, from strict lockdown and semi-lockdown to minimum pandemic-related restrictions, the questionnaire was administered online for people in the community and by health professionals in healthcare facilities. The research was proposed via a link with access to the questionnaire sent by e-mail to people living in the community through the involvement of voluntary associations and neighborhood networks. In healthcare facilities, the researchers met the health professionals willing to co-operate and explained to them the aims and methodology and gave indications on how to submit the survey to participants. Before starting to complete the questionnaires, all participants read the aims of the study, the topics proposed, and the informed consent stating that participation was voluntary and that they could withdraw at any time during the survey. The completion of the questionnaires was anonymous and lasted on average 30 min per participant. The survey consisted of a sociodemographic section focused on different aspects of personal characteristics, lifestyles, social relationships, and four validated questionnaires on the physical and psychological status of health, anxiety and depression, loneliness, and spirituality. The procedures were approved by the local Ethics Committee of the University of Udine and were in accordance with the Helsinki Declaration guidelines.

### 2.2. Participants

A total of 400 older people was firstly contacted to participate in the study, both within the community and in healthcare facilities. Six of these structures were contacted, including nursing homes, residential facilities, and long-term care facilities in the city of Udine. In these facilities, we conducted the research thanks to the co-operation of health professionals which helped the researchers in recruiting participants and in submitting the survey. Overall, we excluded people who were diagnosed with severe psychiatric or neurodegenerative conditions and people who refused to take part in the survey (94 participants) and people over 90 years of age (24 participants). The inclusion of these last subgroups of participants would have made difficult any comparison with the previous literature and normative data. Our final sample was composed of 282 respondents aged 65–89, including both home-dwelling and institutionalized elderly people (76.6% and 23.4% of the total sample, respectively) coming from both urban and rural areas; 163 were women and 119 were men (57.8% and 42.2% of the total sample, respectively).

### 2.3. Measures

#### 2.3.1. Sociodemographic Questionnaire

A sociodemographic questionnaire was developed for the purpose of this study, adapted from the questionnaire used in the study by Crescentini et al. [14] and Feruglio et al. [69]. The first part included some demographic questions (10 items) about participants’ age (possible answers: between 65 and 74 years or between 75 and 89 years), sex, nationality, level of education, marital status, job (or job before retirement), and the number of people with whom they were living. The second group of questions (8 items) focused on changes in their lifestyles during the pandemic, e.g., the amount of time they spent every day practicing physical or recreational activities (range of possible answers: 0–>6 h per day) and if the pandemic modified these habits (increased, decreased, interrupted, or remained the same), and how often they left home during the pandemic (never to every day). The third group of questions (4 items) regarded participants’ family and social network before and during the pandemic: how good they evaluated their relationships with family and friends to be, how often they used to meet their friends before the pandemic (never to always), and if the pandemic modified this habit (increased, decreased, interrupted, or remained the same). The fourth group of questions (5 items) regarded their direct experience with the COVID-19 infection: if they had been tested with swab, if they were positive, if they experienced COVID-19 symptoms, how much they feared being infected (no fear—much fear of contracting the virus), and the amount of time they spent inquiring about the pandemic in the media since COVID-19 breakdown in China on January 2020 (<1–>2 h per day).

#### 2.3.2. Short-Form Health Survey 36 (SF-36)

The SF-36 questionnaire was used to measure the health-related quality of life (HRQOL), being the most popular generic health status measure used in research, due to its comprehensiveness, shortness, and high levels of reliability and validity [70,71,72]. The SF-36 contains 36 questions, which take, on average, 10 min to be answered. It includes 8 health concepts and subscales: physical functioning (PF), role limitations due to physical problems (RP), bodily pain (BP), general health perceptions (GH), vitality (VT), social functioning (SF), role limitations due to emotional problems (RE), and mental health (MH). Each concept is assessed by using a multi-item scale; items scores are summed for each scale and transformed on a scale of 0 to 100, so that higher scores represent better health. The eight subscales design a health profile which is a useful and intuitive tool to describe the HRQOL of a sample. It is also possible to calculate two summary measures which reassume the two major domains of the SF-36: the physical component summary (PSC-36) and the mental component summary (MCS-36). In the present study, as a global measure of health-related quality of life, we considered only the total SF-36 score in the analysis. The Italian version of the SF-36 was elaborated and validated in the IQOLA project, at the end of which it was tested in a large representative sample of the Italian general population (N = 2031) [73]. Cronbach’s alpha for the SF-36 total score in the present study was 0.95.

#### 2.3.3. UCLA Loneliness Scale—Version 3 (UCLA)

The UCLA is a 20-item scale designed to measure one’s subjective feelings of loneliness, as well as feelings of social isolation. Participants rate each item on a scale from 1 (never) to 4 (often). Version 3 [74] is a revised version of both the original UCLA (1978) [75] and the Revised UCLA [76]. The original measure aimed at developing a simple and reliable assessment technique to facilitate the research on loneliness. However, the initial tool was mainly used with college students’ samples, but the reliability of the measure decreased when the scale was used to assess loneliness among other populations, such as the elderly [77]; for this reason, we used version 3 of the scale. Most research on loneliness has been based on the UCLA which has become the “standard” scale in the area (see discussion by Shaver and Brennan [78]). Cronbach’s alpha for the UCLA score in the present study was 0.89.

#### 2.3.4. Hospital Anxiety and Depression Scale (HADS)

The HADS is a frequently used self-rating scale developed to assess psychological distress in non-psychiatric patients (e.g., cancer, coronary heart disease, etc.). It consists of two subscales, Anxiety and Depression [79]. The HADS scale consists of 14 items, 7 for the anxiety subscale (HADS Anxiety) and 7 for the depression subscale (HADS Depression). HADS Anxiety is focused mainly on symptoms of generalized anxiety disorder and HADS Depression is focused on anhedonia, the main symptom of depression. Each item is scored on a response-scale with 4 alternatives ranging between 0 and 3. Overall, it has demonstrated satisfactory psychometric properties in different groups: in primary care patients [80], cognitively intact nursing home patients [81], cancer inpatients [82], and in general populations [79,83]. Djukanovic et al. [84] demonstrated that the HADS scale can be recommended to assess psychological distress among a general population of 65–80 years old, with acceptable internal consistency. Iani et al. [83] confirmed that the HADS has good psychometric properties in an Italian community sample, and that the HADS scores, especially the general psychological distress one, can be reliably used for assessing age and gender differences. Cronbach’s alphas for the HADS Anxiety and Depression scores in the present study were 0.82 and 0.80, respectively.

#### 2.3.5. Functional Assessment of Chronic Illness Therapy—Spiritual Well-Being (FACIT-Sp)

The FACIT-Sp is the most widely used instrument in research to measure spiritual well-being. It is a 12-item scale. Answers are scored on a 5-point Likert scale from 0 to 4. Total scores range from 0 to 48, with higher scores indicating higher spiritual well-being. High spiritual well-being was defined as a FACIT-Sp total score ≥ 36, as proposed by McClain [85], considering the labels corresponding to the scores: a score of 3 on the Likert scale indicates “quite a bit”, while scores below this indicate “somewhat” or lower. The FACIT-Sp has been translated into Italian by the FACIT Organization, using a process of translation-back translation (http://www.facit.org (accessed on 15 January 2021)). We used this tool, although some previous studies [86] suggest that the FACIT-Sp may underestimate spiritual well-being in older patients and, despite having acceptable psychometric properties, may present some limitations for measurement of spiritual well-being in hospitalized elderly patients. Cronbach’s alpha for the FACIT-Sp total score in the present study was 0.86.

### 2.4. Statistical Analysis

Statistical analysis was performed by using R version 3.6.3. Relationships between the measures obtained with the study questionnaires (SF-36, UCLA, FACIT-Sp, HADS Anxiety, and HADS Depression) were performed with Pearson product-moment correlation coefficients. Predictors of the three main study outcomes (HADS Anxiety, HADS Depression, and SF-36 scores) were examined by means of three separate forced-entry multiple regression analyses, using both categorical and continuous variables. Categorical variables (dichotomized as shown in Table 1) that were included in all the three regression models were age, sex, residence, frequency of leaving home in the previous two weeks, change in the amount of physical activity from before to during the pandemic, change in the amount of recreational activities from before to during the pandemic, change in the amount of meetings with family/friends from before to during the pandemic, and the level of fear of COVID-19. Continuous variables that were included in the regression models for HADS Anxiety and HADS Depression scores were the UCLA, FACIT-Sp, and SF-36 scores. Continuous variables that were included in the regression model for SF-36 scores were the UCLA, FACIT-Sp, HADS Anxiety, and HADS Depression scores.

## 3. Results

The study sample consisted of 163 women and 119 men (57.8% and 42.2%); 171 of them were between 65 and 74 years old (57.8%), and 111 were between 75 and 89 years old (60.6% vs. 39.4%). The sample consisted of 216 older people living in their own home (76.6%) and 66 people living in nursing homes (23.4%). Most of the participants were married (56.4%) or widowed (29.8%), and the remaining were single or divorced. The most frequent level of education was high school (41.8%), and the most frequent job before retirement was employee (43.6%). Most of the sample lived alone (44.7%) or with another person (40.4%). As to health problems, more than half of the participants declared not to have any problem (55.3%). As to their social network, most of the participants judged their relationships to be excellent/good (67.0% compared to 33.0% who considered it poor/very bad) and declared that they frequently met with their family and friends (often/always 74.1% vs. rarely/sometimes 25.9%), but during the pandemic, most of them were forced to drastically reduce or interrupt their contacts (78.4% reduced/interrupted vs. 21.6% unchanged/increased). We also considered if they had taken swabs to check for contagion (55.3% answered yes), but few of them resulted in being positive (14.5%) or had symptoms (12.4%). We explored also how much time they spent per day reading or watching the news about the health emergency on TV, newspapers, or the internet: the majority spent less than 2 h per day (85.1%). As to the participants’ daily life during the pandemic, we investigated how often they went out in the previous two weeks (never/sometimes at 49.6% vs. often/daily at 50.4%) and the changes that occurred due to the COVID-19 outbreak in their pre-pandemic habitual physical, social, and recreational activities. As to physical activity, 48.2% of the participants reported 0–2 h of physical activity per day, while the remaining 51.8% reported >2 h of physical activity per day; most of them maintained or improved their practice during the pandemic (61.7% unchanged/increased vs. 38.3% diminished/interrupted). The same can be said for recreational activity: 29.4% of the participants reported 0–2 h of recreational activity per day, while 70.6% reported >2 h of recreational activity per day; this habit was also mostly maintained or improved (84% unchanged/increased vs. 16% diminished/interrupted). Finally, we explored their fear to be infected: most of them were moderately or much afraid (72%), while the others were somewhat or not afraid at all (28%) of being infected. The characteristics of the study sample for the variables included in the regression models are shown in Table 1.

**Figure 1:**
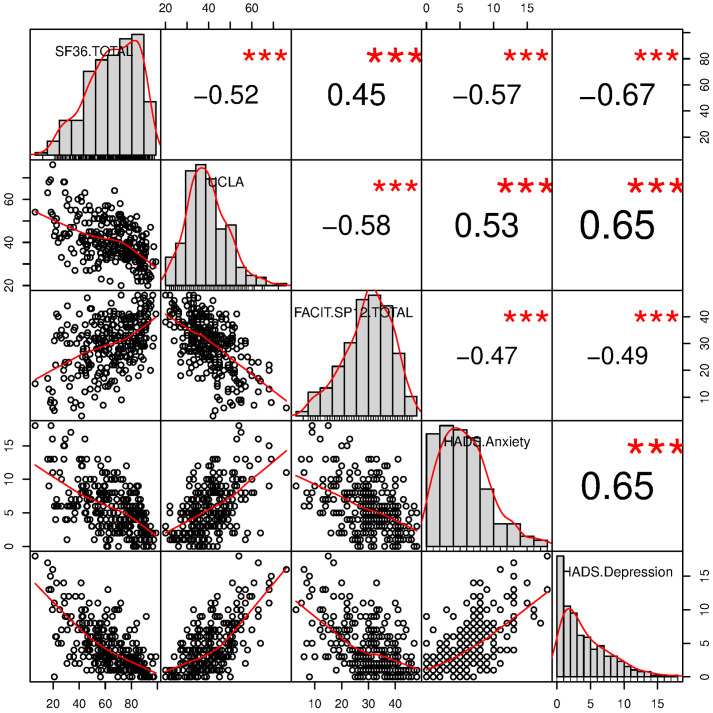
Correlation matrix of the study measures (SF-36, UCLA, FACIT-Sp, HADS Anxiety, and HADS Depression). Asterisks denote a significant correlation, *** *p* < 0.001.

Descriptive statistics of the measures obtained with the study questionnaires (SF-36, UCLA, FACIT-Sp, and HADS), both for the whole sample and for the sample partitioned according to the study variables included in the regression analysis, are shown in Table 2 and Table 3. As portrayed in Figure 1, the distribution of scores was positively skewed for UCLA and HADS (with more frequent scores for the lower levels of loneliness and anxiety/depression) and negatively skewed for SF-36 and FACIT-Sp (with more frequent scores for the higher levels of health-related quality of life and spiritual well-being; for all, *p* < 0.005 in the Shapiro–Wilk normality test). Scores found in the present study on the four questionnaires were generally similar (i.e., remaining within 1 standard deviation from the mean) to those obtained on elderly samples before the pandemic (UCLA: M = 38.6 ± 8.7 in Adams et al. [87]; FACIT-SP: M = 29.6 ± 7.8 in Monod et al. [86]; HADS Anxiety: M = 8.0 ± 4.5, HADS Depression: M = 6.3 ± 4.1 in Iani et al. [83]), except for SF-36 scores, which, in the present study, were lower than those found in the general population in the validation study in Italy (SF-36: M = 73.0 ± 7.7 in Apolone et al. [88]), but higher than those found in a more recent study on older people in Italy (SF-36: M = 48.5 ± 8.8 in Gatti et al. [89]).

The correlation matrix of the measures obtained with the study questionnaires (SF-36, UCLA, FACIT-Sp, and HADS) is shown in Table 3 and Figure 1. The correlation analyses revealed that HADS Anxiety and Depression scores were positively associated with UCLA scores (r = 0.53 and r = 0.65 respectively, for both *p* < 0.001) and negatively associated with FACIT-Sp scores (r = −0.47 and r = −0.49, respectively, for both *p* < 0.001) and SF-36 scores (r = −0.57 and r = −0.67, respectively, for both *p* < 0.001). SF-36 scores, in turn, were positively associated with FACIT-Sp scores (r = 0.45, *p* < 0.001) and negatively associated with UCLA scores (r = −0.52, *p* < 0.001). Finally, UCLA scores were negatively associated with FACIT-Sp scores (r = −0.58, *p* < 0.001).

The three multiple regression analyses that evaluated the predictors of anxiety (HADS Anxiety score), depression (HADS Depression score), and health-related quality of life (SF-36 score) met the assumptions of no perfect multicollinearity (all Variance Inflation Factors, VIFs, were between 1.06 and 2.35) and independence of errors (all Durban–Watson statistics between 1.83 and 2.09). The three models showed that the three sets of predictor variables were significant contributors to the models (see Table 4). The models of HADS Anxiety and HADS Depression explained 50.0% and 59.8% of the variance in HADS Anxiety and HADS depression scores, respectively (R^2^ = 0.500, F (11, 270) = 24.5, *p* < 0.001 for HADS Anxiety; R^2^ = 0.598, F (11, 270) = 36.5, *p* < 0.001 for HADS Depression). The model of SF-36 scores explained 57.7% of the variance in SF-36 scores (R^2^ = 0.577, F (12, 269) = 30.6, *p* < 0.001). In particular, HADS Anxiety levels were predicted by interrupted or diminished meetings with family/friends during the pandemic (β = −0.10, *p* = 0.026), higher fear of COVID-19 (β = 0.15, *p* = 0.002), lower SF-36 scores (β = −0.39, *p* < 0.001), lower FACIT-Sp scores (β = −0.18, *p* < 0.001), and higher UCLA scores (β = 0.25, *p* < 0.001). Interrupted or diminished physical activity during the pandemic was a marginally significant predictor of HADS Anxiety scores (β = −0.09, *p* = 0.058), and a trend was observed for the age variable in predicting HADS Anxiety scores (β = −0.09, *p* = 0.066, younger individuals with higher scores than older ones). HADS Depression levels were instead predicted by higher UCLA scores (β = 0.38, *p* < 0.001) and lower SF-36 scores (β = −0.08, *p* < 0.001). Finally, health-related quality-of-life SF-36 scores were predicted by lower age (β = −0.13, *p* = 0.004), having gone out of the home often or daily in the two weeks before completing the survey (β = 0.18, *p* < 0.001), and by lower HADS Anxiety and Depression scores (β = −0.23 and β = −0.40, respectively, for both *p* < 0.001).

## 4. Discussion

This study investigated a sample of older people (65–89 years old) during the third wave of COVID-19 in Italy, with the aim of exploring the contributing factors of their mental health in relation to the pandemic. A structured survey was designed and administered to both home-dwelling and institutionalized elderly people (76.6% and 23.4% of the total sample, respectively), to both women and men (57.8% and 42.2% of the total sample, respectively), in order to provide a comprehensive picture of the mental health conditions in this age group. This survey was composed of a sociodemographic section (focused on different aspects of personal characteristics, lifestyles, and social relationships) and four validated questionnaires on the health-related quality of life (SF-36), loneliness (UCLA), spirituality (FACIT-Sp), and anxiety/depression (HADS). Each measure was filled in by participants with reference to the last two weeks of their life during spring/summer 2021.

The sociodemographic section of the survey revealed that 49.6% of the elderly included in the study usually remained all day in their home/institution, which appears to be much higher than the pre-pandemic picture in the same age group [90]. This could probably be related to the fact that 72% of the total sample of participants declared a moderate or high fear of COVID-19, which is a much higher value than that obtained in other Italian elderly people during the pandemic: for example, in a survey on more than 20,000 people older than 60 years in April–June 2020, it was found that 61.0% of participants did not fear COVID-19 for themselves and 43.1% did not fear COVID-19 for their family members [91]. The sociodemographic section of the survey of the present study also provided some insightful information on the impact of the pandemic on older people’s lifestyles, which was seen to be worse for the social and physical aspects of daily life than for the recreational aspects. Indeed, only 16.0% of the respondents declared that the COVID-19 pandemic caused an interruption/diminishment of their usual pre-pandemic recreational activities, but a higher number of them declared that the COVID-19 outbreak caused an interruption/diminishment of their usual pre-pandemic physical activities (38.3%) and of the meetings with their family and friends (78.4%). These data should be interpreted while bearing in mind the pre-pandemic lifestyle of Italian elderly vs. citizens of other European states: in comparison with European people older than 65 years in 2019, the Italian elderly showed below-average levels in participation in cultural/sporting events and in tourism and of physical activity, while above-average levels were reported with reference to frequency of contacts with family, relatives, or friends [92].

The second part of the survey consisted of four validated questionnaires on health-related quality of life, loneliness, spirituality, and anxiety/depression. The scores obtained with these four measures were all related to each other in the expected direction [93,94,95,96,97]: for example, health-related quality of life and spirituality were positively associated to each other, and so were loneliness and anxiety/depression scores; moreover, the first pair of variables was negatively associated to the second pair of variables. Furthermore, the data obtained in these questionnaires revealed that the scores obtained by our sample of Italian elderly during the third wave of the COVID-19 pandemic in 2021 were generally in line with those obtained on elderly samples before the pandemic [83,86,87]. This suggests that the psychological and spiritual well-being of the elderly sample studied in the present research had not been seriously impaired by the events related to the COVID-19 pandemic. Although elderly people have been affected by the pandemic in some way since 2020 in terms of loneliness or emotional well-being [98,99,100,101], several other studies highlighted that older people reported levels of loneliness, distress, and well-being that appeared to be less deteriorated and more stable in time than those provided by young and middle-aged respondents [102,103,104,105,106,107,108]. This could also be due to the fact that we investigated the sample during the third COVID-19 pandemic wave and individuals could have had the possibility to become more resilient. In fact, other previous research has shown that the increase in mental health symptoms was largest among studies that sampled participants in the early stages of the pandemic (March–April 2020), but then their severity decreased significantly over the following months (May–July 2020) [109]. This pattern may represent an acute and normal response to an unforeseen and distressing traumatic event [110], which was then followed by a period of psychological adaptation and resilience [111,112,113]. Similarly, in a large sample of UK adults recruited after the pandemic outbreak in 2020, both anxiety and depressive symptoms showed a trajectory of recovery from the beginning of April 2020 onward [114].

In addition to the descriptive and correlational analyses of the data obtained from the survey, the present study tried to identify the predictors of the levels of anxiety, depression, and health-related quality of life of elderly participants by means of three multiple regression models. As predictors of these models, a selection of variables employed in the sociodemographic section and the loneliness and spirituality scores were used, as well as the health-related quality-of-life scores in the models for anxiety and depression, and the anxiety and depression scores in the model for health-related quality of life. For all three models, predictors explained more than half of the variance of the respective outcome. In particular, the model for anxiety revealed that higher anxiety scores were predicted by lower quality-of-life and spirituality scores, as well as by higher loneliness and fear-of-COVID-19 scores. Older people’s anxiety levels were also positively predicted by the interruption/diminishment of their usual pre-pandemic meetings with family and friends. Of great importance, this latter result confirms the findings obtained in other studies on elderly samples conducted before and after the COVID-19 outbreak [34,115] and highlights the importance of social support for elderly for the mitigation of their anxiety levels, in particular for programming interventions in future pandemic-like emergencies.

In the model for depression, the changes in social meetings with family/friends or in physical or recreational activities did not emerge as significant predictors of depression. However, loneliness scores, which are ordinarily related to the sense of social connectedness, remained a strong predictor of depression, as it was for anxiety. As to depression, the other significant predictor was the score in health-related quality of life, with a negative relationship, as it was for anxiety. Quite surprisingly, in the two regression models for anxiety and depression, the variables of sex, age group (65–74 vs. 75–89 years), and residence (one’s own house or institution) did not emerge as significant predictors. Research has indeed shown that women generally experience higher levels of anxiety and depression than men in the older age groups [83,116,117,118,119,120], that depression levels generally increase with age in the elderly [117,121,122], and that depression levels are usually higher in institutionalized than in community-dwelling older adults [123,124,125]. However, this previous research refers to pre-pandemic conditions and should be confirmed and validated by further data collected during the actual COVID-19 and other similar public-health emergencies.

In the model for health-related quality of life, participant’s age group instead emerged as a significant predictor, with older age being associated with worse quality-of-life scores. The difference between the two age groups in terms of health-related quality of life can also be plausibly due to the fact that the SF-36 questionnaire used for measuring health-related quality of life contains a consistent number of items on the health status of the respondent, which is typically worse in older than younger individuals. Anxiety and depression scores emerged as significant predictors of health-related quality of life, as well, clearly with a negative mutual relationship. Finally, another variable turned out to be a significant predictor of participants’ quality of life, namely the attitude toward remaining at home or in the institution all day rather than going out for a walk, a visit, or another reason: the elderly people who usually remained in their home/institution all day self-reported a worse health-related quality of life than those who usually or daily left their home/institution. This specific variable (going out or remaining at home) has not been studied in depth in relation to the quality of life of the elderly during the pandemic; thus, future studies may further consider this aspect [126].

In conclusion, the present study carried out on older Italian individuals during the third wave of the pandemic found largely preserved levels of emotional functioning, perceived loneliness, and quality of life. This overall result emerged despite the fact that the large majority of participants reported a worsening of their social (more than physical and recreational) life and a moderate-to-high fear of COVID-19 (apparently in a larger proportion than in other studies on the elderly). In regression analyses, these two latter aspects turned out to be significant predictors of higher anxiety levels in the present sample. Spiritual well-being was also similar to pre-pandemic levels and emerged as a significant protective factor against anxiety symptoms. Finally, the possibility going on excursions away from the home/institution arose as the most important lifestyle factor for preserving the quality of life of the elderly.

Some limitations should be acknowledged in the present study. One limit concerns data collection and analysis, as it could have been impacted by the potential presence of mild dementia in older participants. As we could not accurately assess mild dementia in our sample, future studies could explore how similar measures of health-related quality of life, anxiety, and depression could be impacted by this clinical condition during pandemic periods (e.g., Reference [127]). Another limit of the study pertains to the choice of carrying out forced-entry regressions, where all variables are entered in the model simultaneously. This allowed us to give the same priority to all study variables, but, given the quite large number of predictors and the significant correlation between the questionnaire scores, it did not allow us to test the influence of a more restricted set of variables (e.g., the sociodemographic items only) on the three model outcomes.

## 5. Conclusions

The information provided by this study helps refine the picture of the current condition of the elderly in our society in the aftermath of the COVID-19 pandemic, thus giving some hints as to how to continue supporting their mental health and quality of life. Several actions can be implemented based on the WHO Active Ageing Policy, adopted by many countries, which focuses on health promotion and protection. In particular, as regards social isolation and loneliness, anxiety, and depression, among older people, many interventions and strategies at the individual-, relationship-, and community-level have shown promise, including the promotion of healthy lifestyles and spirituality; however, evidence on how well they work is yet very limited, and further research is needed [47].

## Figures and Tables

**Table 1:** Characteristics of study participants (N = 282).

Variable	N	%
Sex		
female	163	57.8
male	119	42.2
Age		
65–74 years	171	60.6
75–89 years	111	39.4
Residence		
in own home	216	76.6
in nursing home	66	23.4
Frequency of leaving home in the previous 2 weeks		
never or sometimes	140	49.6
often or daily	142	50.4
Physical activity during the pandemic		
interrupted or diminished	108	38.3
unchanged or increased	174	61.7
Recreational activities during the pandemic		
interrupted or diminished	45	16.0
unchanged or increased	237	84.0
Meetings with family/friends during the pandemic		
interrupted or diminished	221	78.4
unchanged or increased	61	21.6
Fear of COVID-19		
none or low	79	28.0
moderate or high	203	72.0

**Table 2:** Mean and standard deviation scores of the questionnaires employed in the study by function of the main study variables. * Asterisks denote a significant difference (*p* < 0.01 obtained after Bonferroni correction for multiple comparisons across the five questionnaires) in the questionnaire means obtained from the two levels of each study variable.

Variable Level	SF-36		UCLA		FACIT-Sp		HADS Anxiety		HADS Depression	
Sex										
female	61.5 ± 20.3		41.4 ± 10.5		29.4 ± 10.1		6.3 ± 3.7		4.7 ± 3.7	
male	67.5 ± 20.5		39.7 ± 9.0		30.5 ± 9.1		5.3 ± 3.7		4.0 ± 3.6	
Age										
65–74 years	68.1 ± 19.7	*	39.5 ± 9.7		30.7 ± 9.3		5.9 ± 3.6		4.0 ± 3.5	
75–89 years	57.7 ± 20.4		42.5 ± 9.9		28.6 ± 10.1		5.8 ± 4.0		4.9 ± 3.8	
Residence										
home-dwelling	67.2 ± 19.7	*	39.6 ± 9.6	*	30.5 ± 8.9		5.8 ± 3.8		4.1 ± 3.6	
nursing home	53.8 ± 20.2		44.2 ± 10.0		27.9 ± 11.7		6.0 ± 3.7		5.3 ± 3.7	
Frequency of leaving home in the previous 2 weeks										
never or sometimes	55.9 ± 21.3	*	43.1 ± 10.7	*	28.1 ± 10.8	*	6.5 ± 4.0	*	5.2 ± 4.1	*
often or daily	72.1 ± 16.3		38.3 ± 8.4		31.7 ± 8.0		5.2 ± 3.3		3.5 ± 3.0	
Physical activity during the pandemic										
interrupted or diminished	63.1 ± 19.0		41.5 ± 9.5		30.3 ± 8.9		6.5 ± 3.9		4.7 ± 3.6	
unchanged or increased	64.7 ± 21.5		40.2 ± 10.2		29.6 ± 10.2		5.4 ± 3.6		4.1 ± 3.6	
Recreational activities during the pandemic										
interrupted or diminished	52.8 ± 21.9	*	44.8 ± 8.0	*	28.6 ± 8.5		7.2 ± 4.0		6.3 ± 4.2	*
unchanged or increased	66.2 ± 19.6		39.9 ± 10.0		30.1 ± 9.9		5.6 ± 3.6		4.0 ± 3.4	
Meetings with family/friends during the pandemic										
interrupted or diminished	63.7 ± 20.5		40.5 ± 9.9		30.1 ± 9.4		6.1 ± 3.7		4.5 ± 3.7	
unchanged or increased	65.4 ± 21.0		41.2 ± 10.0		29.0 ± 10.6		4.9 ± 3.6		4.0 ± 3.4	
Fear of COVID-19										
none or low	66.0 ± 20.5		41.0 ± 10.9		29.8 ± 10.7		4.5 ± 3.5	*	3.7 ± 3.6	
moderate or high	63.3 ± 20.6		40.6 ± 9.5		29.9 ± 9.3		6.3 ± 3.7		4.6 ± 3.7	

**Table 3:** Descriptive statistics of the questionnaires employed, both for the present study and for reference studies.

Questionnaire	Present Study M ± SD	Reference Study M ± SD
SF-36	64.0 ± 20.6	73.0 ± 7.7 [88] 48.5 ± 8.8 [89]
UCLA	40.7 ± 9.9	38.6 ± 8.7 [87]
FACIT-SP12	29.9 ± 9.7	29.6 ± 7.8 [86]
HADS Anxiety	5.8 ± 3.7	8.0 ± 4.5 [83]
HADS Depression	4.4 ± 3.6	6.3 ± 4.1 [83]

**Table 4:** Multiple linear regression models of anxiety symptoms (HADS Anxiety score), depression symptoms (HADS Depression score), and health-related quality of life (SF-36 score); * *p* < 0.05, ** *p* < 0.01, and *** *p* < 0.001.

	HADS Anxiety Score			HADS Depression Score			SF-36 Total Score		
Predictor	B (SE)	β	*p*	B (SE)	β	*p*	B (SE)	β	*p*
Sex (0 = female, 1 = male)	−0.30 (0.33)	−0.04	0.374	0.09 (0.29)	0.01	0.766	2.10 (1.70)	0.05	0.216
Age (0 = 65–74 years; 1 = 75–89 years)	−0.68 (0.37)	−0.09	0.066	−0.20 (0.32)	−0.03	0.530	−5.35 (1.84)	−0.13	0.004 **
Residence (0 = home-dwelling; 1 = nursing home)	−0.55 (0.48)	−0.06	0.250	−0.08 (0.42)	−0.01	0.856	−3.51 (2.43)	−0.07	0.151
Frequency of leaving home in the previous 2 weeks (0 = never or sometimes; 1 = often or daily)	0.14 (0.39)	0.02	0.728	0.35 (0.34)	0.05	0.301	7.24 (1.94)	0.18	<0.001 ***
Physical activity during the pandemic (0 = interrupted or diminished; 1 = unchanged or increased)	−0.66 (0.35)	−0.09	0.058	−0.20 (0.30)	−0.03	0.519	−1.48 (1.77)	−0.04	0.401
Recreational activities during the pandemic (0 = interrupted or diminished; 1 = unchanged or increased)	0.14 (0.47)	0.01	0.762	−0.51 (0.41)	−0.05	0.222	2.95 (2.40)	0.05	0.220
Meetings with family/friends during the pandemic (0 = interrupted or diminished; 1 = unchanged or increased)	−0.91 (0.41)	−0.10	0.026 *	−0.28 (0.36)	−0.03	0.432	−0.99 (2.09)	−0.02	0.638
Fear of COVID-19 (0 = none or low; 1 = moderate or high)	1.22 (0.39)	0.15	0.002 **	0.56 (0.34)	0.07	0.103	−2.13 (2.01)	−0.05	0.290
SF-36 Total score	−0.07 (0.01)	−0.39	<0.001 ***	−0.08 (0.01)	−0.45	<0.001 ***	−	−	−
UCLA score	0.09 (0.02)	0.25	<0.001 ***	0.14 (0.02)	0.38	<0.001 ***	−0.01 (0.12)	−0.00	0.949
FACIT-Sp score	−0.07 (0.02)	−0.18	0.001 **	−0.03 (0.02)	−0.08	0.088	0.18 (0.11)	0.08	0.108
HADS Anxiety score	−	−	−	−	−	−	−1.24 (0.31)	−0.23	<0.001 ***
HADS Depression score	−	−	−	−	−	−	−2.24 (0.34)	−0.40	<0.001 ***
	R^2^ = 0.500			R^2^ = 0.598			R^2^ = 0.577		
	adjusted R^2^ = 0.479			adjusted R^2^ = 0.582			adjusted R^2^ = 0.559		
	*p* < 0.001 ***			*p* < 0.001 ***			*p* < 0.001 ***		

## Data Availability

Raw data supporting the conclusions of this article will be made available by the authors upon request, without undue reservation.

## References

[1] Mehra A., Rani S., Sahoo S., Parveen S., Singh A.P., Chakrabarti S., Grover S. (2020). A crisis for elderly with mental disorders: Relapse of symptoms due to heightened anxiety due to COVID-19. Asian J. Psychiatr..

[2] Ho C.S., Chee C.Y., Ho R.C. (2020). Mental Health Strategies to Combat the Psychological Impact of Coronavirus Disease 2019 (COVID-19) Beyond Paranoia and Panic. Ann. Acad. Med. Singap..

[3] World Health Organization (2020). World Health Organization: Mental Health and Psychosocial Considerations During COVID-19 Outbreak. https://www.who.int/docs/default-source/coronaviruse/mental-health-considerations.pdf.

[4] Cacioppo J.T., Cacioppo S. (2018). Loneliness in the Modern Age: An Evolutionary Theory of Loneliness (ETL).

[5] World Health Organization (2020). Coronavirus. https://www.who.int/health-topics/coronavirus#tab=tab_1.

[6] Manchia M., Gathier A.W., Yapici-Eser H., Schmidt M.V., de Quervain D., van Amelsvoort T., Bisson J.I., Cryan J.F., Howes D.O., Pinto L. (2022). The impact of the prolonged COVID-19 pandemic on stress resilience and mental health: A critical review across waves. Eur. Neuropsychopharmacol..

[7] Verity R., Okell L.C., Dorigatti I., Winskill P., Whittaker C., Imai N., Cuomo-Dannenburg G., Thompson H., Walker P.G.T., Fu H. (2020). Estimates of the severity of coronavirus disease 2019: A model-based analysis. Lancet Infect. Dis..

[8] Armitage R., Nellums L.B. (2020). COVID-19 and the consequences of isolating the elderly. Lancet Public Health.

[9] Galea S., Merchant R.M., Lurie N. (2020). The mental health consequences of COV_ID-19 and physical distancing: The need for prevention and early ıntervention. JAMA Intern. Med..

[10] Steinman M.A., Perry L., Perissinotto C.M. (2020). Meeting the care needs of older adults ısolated at home during the COVID-19 pandemic. JAMA Int. Med..

[11] Fegert J.M., Vitiello B., Plener P.L., Clemens V. (2020). Challenges and burden of the Coronavirus 2019 (COVID-19) pandemic for child and adolescent mental health: A narrative review to highlight clinical and research needs in the acute phase and the long return to normality. Child Adolesc. Psychiatry Ment. Health.

[12] Holmes E.A., O’Connor R.C., Perry V.H., Tracey I., Wessely S., Arseneault L., Ballard C., Christensen H., Silver R.C., Everall I. (2020). Multidisciplinary research priorities for the COVID-19 pandemic: A call for action for mental health science. Lancet Psychiatry.

[13] Matiz A., Fabbro F., Paschetto A., Urgesi C., Ciucci E., Baroncelli A., Crescentini C. (2022). The impact of the COVID-19 pandemic on affect, fear, and personality of primary school children measured during the second wave of infections in 2020. Front. Psychiatry.

[14] Crescentini C., Feruglio S., Matiz A., Paschetto A., Vidal E., Cogo P., Fabbro F. (2020). Stuck Outside and Inside: An Exploratory Study on the Effects of the COVID-19 Outbreak on Italian Parents and Children’s Internalizing Symptoms. Front. Psychol..

[15] Eurofound (2021). Impact of COVID-19 on Young People in the EU.

[16] Bassi F., Doria M. (2022). Diffusion of COVID-19 among children and adolescents during the second and third waves of the pandemic in Italy. Eur. J. Pediatr..

[17] Doraiswamy S., Mamtani R., Ameduri M., Abraham A., Cheema S. (2020). Respiratory epidemics and older people. Age Ageing.

[18] Gerlach L., Solway E., Singer D., Kullgren J., Kirch M., Malani P. (2021). Mental Health among Older Adults before and during the COVID-19 Pandemic. University of Michigan National Poll on Healthy Aging. https://www.healthyagingpoll.org/reports-more/report/mental-health-among-older-adults-and-during-covid-19-pandemic.

[19] Delam H., Izanloo S. (2020). Increased death anxiety in the elderly during coronavirus disease 2019 (COVID-19) pandemic. J. Health Sci. Surveill. Syst..

[20] Wang C., Pan R., Wan X., Tan Y., Xu L., Ho C.S., Ho R.C. (2020). Immediate psychological responses and associated factors during the initial 20 OMEGA—Journal of Death and Dying 0 stage of the 2019 coronavirus disease (COVID-19) epidemic among the general population in China. Int. J. Environ. Res. Public Health.

[21] Bala R., Maheshwari S.K. (2018). Death anxiety and death depression among elderly. Int. J. Psychiatr. Nurs..

[22] Khademi F., Moayedi S., Golitaleb M., Karbalaie N. (2021). Letter to the edıtor. The COVID-19 pandemic and death anxiety in the elderly. Int. J. Ment. Health Nurs..

[23] Meng H., Xu Y., Dai J., Zhang Y., Liu B., Yang H. (2020). The psychological effect of COVID-19 on the elderly in China. Psychiatry Res..

[24] Vahia I.V., Jeste D.V., Reynolds C.F. (2020). III. Older adults and the mental health effects of COVID-19. JAMA.

[25] Das S., Arun P., Rohilla R., Parashar K., Roy A. (2021). Anxiety and depression in the elderly due to COVID-19 pandemic: A pilot study. Middle East Curr. Psychiatry.

[26] Carstensen L.L., Shavit Y.Z., Barnes J.T. (2020). Age Advantages in Emotional Experience Persist Even Under Threat From the COVID-19 Pandemic. Psychol Sci..

[27] Carbone E., Palumbo R., Sella E., Lenti G., Di Domenico A., Borella E. (2021). Emotional, Psychological, and Cognitive Changes Throughout the COVID-19 Pandemic in Italy: Is There an Advantage of Being an Older Adult?. Front. Aging Neurosci..

[28] Ceccato I., Palumbo R., Di Crosta A., La Malva P., Marchetti D., Maiella R., Verrocchio M.C., Marin A., Mammarella N., Palumbo R. (2020). Age-related differences in the perception of COVID-19 emergency during the Italian outbreak. Aging Ment. Health.

[29] Young N.A., Waugh C.E., Minton A.R., Charles S.T., Haase C.M., Mikels J.A. (2021). Reactive, agentic, apathetic, or challenged? Aging, emotion, and coping during the COVID-19 pandemic. Gerontologist.

[30] Machielse A. (2006). Theories on social contacts and social isolation. Hortulanus R, Machielse A, Meeuwesen, L. Social Isolation in Modern Society.

[31] Pugh S., Victor C., Scambler S., Bond J. (2009). The Social World of Older People: Understanding Loneliness and Social Isolation in Later Life (Growing Older).

[32] Nicholson N.R. (2012). A review of social isolation: An important but underassessed condition in older adults. J. Primary Prev..

[33] Gray K. (2019). A Conceptual Review of Loneliness Across the Adult Life Course (16+ Years).

[34] Santini Z.I., Jose P.E., Cornwell E.Y., Koyanagi A., Nielsen L., Hinrichsen C., Meilstrup C., Madsen K.R., Koushede V. (2020). Social disconnectedness, perceived isolation, and symptoms of depression and anxiety among older Americans (NSHAP): A longitudinal mediation analysis. Lancet Public Health.

[35] Das A. (2019). Loneliness does (not) have cardiometabolic effects: A longitudinal study of older adults in two countries. Soc. Sci. Med..

[36] Ong A.D., Uchino B.N., Wethington E. (2016). Loneliness and health in older adults: A mini-review and synthesis. Gerontology.

[37] Evans I.E.M., Martyr A., Collins R., Brayne C., Clare L. (2019). Social isolation and cognitive function in later life: A systematic review and meta-analysis. J. Alzheimers Dis..

[38] Kuiper J.S., Zuidersma M., Oude Voshaar R.C., Zuidema S.U., van den Heuvel E.R., Stolk R.P., Smidt N. (2015). Social relationships and risk of dementia: A systematic review and meta-analysis of longitudinal cohort studies. Ageing Res. Rev..

[39] Lara E., Martín-María N., De la Torre-Luque A., Koyanagi A., Vancampfort D., Izquierdo A., Miret M. (2019). Does loneliness contribute to mild cognitive impairment and dementia? A systematic review and meta-analysis of longitudinal studies. Ageing Res. Rev..

[40] Leigh-Hunt N., Bagguley D., Bash K., Turner V., Turnbull S., Valtorta N., Caan W. (2017). An overview of systematic reviews on the public health consequences of social isolation and loneliness. Public Health.

[41] Dahlberg L. Loneliness during the COVID-19 pandemic. Aging Ment. Health.

[42] UN (2020). Policy Brief: The Impact of COVID-19 on Older Persons.

[43] Ayalon L., Chasteen A., Diehl M., Levy B.R., Neupert S.D., Rothermund K., Tesch-Romer C., Wahl H.W. (2021). Aging in times of the COVID-19 pandemic: Avoiding ageism and fostering intergenerational solidarity. J. Gerontol. Ser. B.

[44] Brooke J., Jackson D. (2020). Older people and COVID-19: Isolation, risk and ageism. J. Clin. Nurs..

[45] Pancani L., Marinucci M., Aureli N., Riva P. (2020). Forced Social Isolation and Mental Health: A Study on 1006 Italians under COVID-19 Quarantine. https://psyarxiv.com/uacfj/download?format=pdf.

[46] Jopling K. (2020). Promising Approaches Revisited: Effective Action on Loneliness in Later Life.

[47] World Health Organization (2021). Global Report on Ageism.

[48] Lu C., Chi X., Liang K., Chen S.T., Huang L., Guo T., Jiao C., Yu Q., Veronese N., Soares F.C. (2020). Moving More and Sitting Less as Healthy Lifestyle Behaviors are Protective Factors for Insomnia, Depression, and Anxiety Among Adolescents During the COVID-19 Pandemic. Psychol. Res. Behav. Manag..

[49] Fullana M.A., Hidalgo-Mazzei D., Vieta E., Radua J. (2020). Coping behaviors associated with decreased anxiety and depressive symptoms during the COVID-19 pandemic and lockdown. J. Affect. Disord..

[50] Ellul M.A., Benjamin L., Singh B., Lant S., Michael B.D., Easton A., Kneen R., Defres S., Sejvar J., Solomon T. (2020). Neurological associations of COVID-19. Lancet Neurol..

[51] Schuch F.B., Vancampfort D., Richards J., Rosenbaum S., Ward P.B., Stubbs B. (2016). Exercise as a treatment for depression: A meta-analysis adjusting for publication bias. J. Psychiatr. Res..

[52] Maugeri G., Castrogiovanni P., Battaglia G., Pippi R., D’Agata V., Palma A., Di Rosa M., Musumeci G. (2020). The impact of physical activity on psychological health during COVID-19 pandemic in Italy. Heliyon.

[53] Lange K.W., Nakamura Y. (2020). Lifestyle factors in the prevention of COVID-19. Global Health J..

[54] Lange K.W. (2018). Diet, Exercise, and Mental Disorders—Review Public Health Challenges of the Future.

[55] Maunder R., Hunter J., Vincent L., Bennett J., Peladeau N., Leszcz M., Sadavoy J., Verhaeghe L.M., Steinberg R., Mazzulli T. (2003). The immediate psychological and occupational impact of the 2003 SARS outbreak in a teaching hospital. CMAJ.

[56] Rogers J.P., Chesney E., Oliver D., Pollak T.A., McGuire P., Fusar-Poli P., Zandi M.S., Lewis G., David A.S. (2020). Psychiatric and neuropsychiatric presentations associated with severe coronavirus infections: A systematic review and meta-analysis with comparison to the COVID-19 pandemic. Lancet Psychiatry.

[57] Gencer N. (2019). Ritualization as alternative approach to the spiritual dimension of palliative care: A concept analysis. J. Relig. Stud..

[58] Klavuz M.A., Klavuz E., Koc A.M., Tınaz N. (2016). The ımportance of spiritual counseling services in coping with losses in the aging period. Spiritual Counseling and Guidance.

[59] Harris G.M., Allen R.S., Dunn L., Parmelee P. (2013). “Trouble won’t last always”: Religious coping and meaning in the stress process. Qual. Health Res..

[60] Rote S., Hill T.D., Ellison C.G. (2013). Religious attendance and loneliness in later life. Gerontologist.

[61] Pink J., Jacobson L., Pritchard M. (2007). The 21st century GP: Physician and priest?. Br. J. Gen. Pract..

[62] Koenig H.G. (2020). Ways of protecting religious older adults from the consequences of COVID-19. Am. J. Geriatr. Psychiatry.

[63] Archbald-Pannone L. (2020). COVID-19: 4 Tips to Help the Elderly Stay Connected. https://www.weforum.org/agenda/2020/03/seniors-elderly-coronavirus-isolation/.

[64] Savci C., Cil Akinci A., Yildirim Usenmez S., Keles F. (2021). The effects of fear of COVID-19, loneliness, and resilience on the quality of life in older adults living in a nursing home. Geriatr. Nurs..

[65] Madden A., Leen B. (2020). Evidence Summary: What is the Impact of the Coronavirus Pandemic on the Mental Health of Elderly Nursing Home Residents?.

[66] Kaelen S., van den Boogaard W., Pellecchia U., Spiers S., De Cramer C., Demaegd G., Fouqueray E., Van den Bergh R., Goublomme S., Decroo T. (2021). How to bring residents’ psychosocial well-being to the heart of the fight against COVID-19 in Belgian nursing homes-A qualitative study. PLoS ONE..

[67] Williams J., Lyons B., Rowland D. (1997). Unmet long-term care needs of elderly people in the community: A review of the literature. Home Health Care Serv. Q.

[68] Shrivastava S.R., Shrivastava P.S., Ramasamy J. (2013). Healthcare of elderly: Determinants, needs and services. Int. J. Prev. Med..

[69] Feruglio S., Matiz A., Cogo P., Vidal E., Paschetto A., Fabbro F., Crescentini C. (2022). Isolated and blocked adolescents: A study on the psychological effects of the COVID-19 outbreak. Minerva Psichiatr..

[70] Ware J.E., Sherbourne C.D. (1992). The MOS 36-item short-form health survey (SF-36). I. Conceptual framework and item selection. Med Care..

[71] McHorney C.A., Ware J.E., Raczek A.E. (1993). The Mos 36-Item Short-Form Health Survey (SF-36): II. Psychometric and clinical tests of validity in measuring physical and mental health constructs. Med. Care.

[72] Ware J.E. (1993). SF-36 Health Survey. Manual and Interpretation Guide.

[73] Ware J.E., Gandek B., Kosinski M., Aaronson N.K., Apolone G., Brazier J.E., Bullinger M., Kaasa S., Leplege A., Prieto L. (1998). The equivalence of SF-36 Summary Health scores estimated using standard and country-specific algorithms in 10 countries: Results from the IQOLA Project. J. Clin. Epidemiol..

[74] Russell D. (1996). UCLA Loneliness Scale (Version 3): Reliability, validity, and factor structure. J. Personal. Assess..

[75] Russell D., Peplau L.A., Ferguson M.L. (1978). Developing a measure of loneliness. J. Personal. Assess..

[76] Russell D., Peplau L.A., Cutrona C.E. (1980). The Revised UCLA Loneliness Scale: Concurrent and discriminate validity evidence. J. Personal. Soc. Psychol..

[77] Cutrona C., Russell D., Rose J. (1986). Social support and adaptation to stress by the elderly. Psychol. Aging.

[78] Shaver P.R., Brennan K.A., Robinson J.P., Shaver P.R., Wrightsman L.S. (1991). Measures of depression and loneliness. Measures of personality and social psychological attitudes.

[79] Zigmond A.S., Snaith R.P. (1983). The hospital anxiety and depression scale. Acta Psychiatr. Scand..

[80] Bjelland I., Dahl A.A., Haug T.T., Neckelmann D. (2002). The validity of the hospital anxiety and depression scale-an updated literature review. J. Psychosom. Res..

[81] Drageset J., Eide G.E., Ranhoff A.H. (2013). Anxiety and depression among nursing home residents without cognitive impairment. Scand. J. Caring Sci..

[82] Annunziata M., Muzzatti B., Altoe G. (2011). Defining hospital anxiety and depression scale (HADS) structure by confirmatory factor analysis: A contribution to validation for oncological settings. Ann. Oncol..

[83] Iani L., Lauriola M., Costantini M. (2014). A confirmatory bifactor analysis of the hospital anxiety and depression scale in an Italian community sample. Health Qual. Life Outcomes.

[84] Djukanovic I., Carlsson J., Årestedt K. (2017). Is the Hospital Anxiety and Depression Scale (HADS) a valid measure in a general population 65-80 years old? A psychometric evaluation study. Health Qual. Life Outcomes.

[85] McClain C.S., Rosenfeld B., Breitbart W. (2003). Effect of Spiritual Well-Being on End-of-Life Despair in Terminally-Ill Cancer Patients. Lancet.

[86] Monod S., Lécureux E., Rochat E., Spencer B., Seematter-Bagnoud L., Martin-Durussel A., Büla C. (2015). Validity of the FACIT-Sp to Assess Spiritual Well-Being in Elderly Patients. Psychology.

[87] Adams K.B., Sanders S., Auth E.A. (2004). Loneliness and depression in independent living retirement communities: Risk and resilience factors. Aging Ment. Health.

[88] Apolone G., Mosconi P. (1998). The Italian SF-36 Health Survey: Translation, validation and norming. J. Clin. Epidemiol..

[89] Gatti A., Gottschling J., Brugnera A., Adorni R., Zarbo C., Compare A., Segal D.L. (2018). An investigation of the psychometric properties of the Geriatric Anxiety Scale (GAS) in an Italian sample of community-dwelling older adults. Aging Ment. Health.

[90] Jacobs J.M., Hammerman-Rozenberg A., Stessman J. (2018). Frequency of Leaving the House and Mortality from Age 70 to 95. J. Am. Geriatr. Soc..

[91] Cori L., Curzio O., Adorni F., Prinelli F., Noale M., Trevisan C., Fortunato L., Giacomelli A., Bianchi F. (2021). Fear of COVID-19 for individuals and family members: Indications from the national cross-sectional study of the epicovid19 web-based survey. Int. J. Environ. Res. Public Health.

[92] EUROSTAT 2020 (2020). Ageing Europe—Looking at the Lives of Older People in the EU—2020 Edition.

[93] Routasalo P., Pitkala K.H. (2003). Loneliness among older people. Rev. Clin. Gerontol..

[94] Luanaigh C.Ó., Lawlor B.A. (2008). Loneliness and the health of older people. Int. J. Geriatr. Psychiatry.

[95] Zimmer Z., Jagger C., Chiu C.T., Ofstedal M.B., Rojo F., Saito Y. (2016). Spirituality, religiosity, aging and health in global perspective: A review. SSM Popul. Health.

[96] Thauvoye E., Vanhooren S., Vandenhoeck A., Dezutter J. (2018). Spirituality and well-being in old age: Exploring the dimensions of spirituality in relation to late-life functioning. J. Relig. Health.

[97] Van As B.A.L., Imbimbo E., Franceschi A., Menesini E., Nocentini A. (2021). The longitudinal association between loneliness and depressive symptoms in the elderly: A systematic review. Int. Psychogeriatr..

[98] Stolz E., Mayerl H., Freidl W. (2021). The impact of COVID-19 restriction measures on loneliness among older adults in Austria. Eur. J. Public Health.

[99] Macdonald B., Hülür G. (2021). Well-being and loneliness in Swiss older adults during the COVID-19 pandemic: The role of social relationships. Gerontologist.

[100] Krendl A.C., Perry B.L. (2021). The impact of sheltering in place during the COVID-19 pandemic on older adults’ social and mental well-being. J. Gerontol. Series B.

[101] Zaninotto P., Iob E., Demakakos P., Steptoe A. (2022). Immediate and longer-term changes in the mental health and well-being of older adults in England during the COVID-19 pandemic. JAMA Psychiatry.

[102] Choi I., Kim J.H., Kim N., Choi E., Choi J., Suk H.W., Na J. (2021). How COVID-19 affected mental well-being: An 11-week trajectories of daily well-being of Koreans amidst COVID-19 by age, gender and region. PLoS ONE.

[103] Bu F., Steptoe A., Fancourt D. (2020). Loneliness during a strict lockdown: Trajectories and predictors during the COVID-19 pandemic in 38,217 United Kingdom adults. Soc. Sci. Med..

[104] Bidzan-Bluma I., Bidzan M., Jurek P., Bidzan L., Knietzsch J., Stueck M., Bidzan M. (2020). A Polish and German population study of quality of life, well-being, and life satisfaction in older adults during the COVID-19 pandemic. Front. Psychiatry.

[105] Jiang D. (2020). Perceived stress and daily well-being during the COVID-19 outbreak: The moderating role of age. Front. Psychol..

[106] O’Connor R.C., Wetherall K., Cleare S., McClelland H., Melson A.J., Niedzwiedz C.L., O’Carroll R.E., O’Connor D.B., Platt S., Scowcroft E. (2021). Mental health and well-being during the COVID-19 pandemic: Longitudinal analyses of adults in the UK COVID-19 Mental Health & Wellbeing study. Br. J. Psychiatry.

[107] Carney A.K., Graf A.S., Hudson G., Wilson E. (2021). Age moderates perceived COVID-19 disruption on well-being. Gerontologist.

[108] Gallagher M.W., Smith L.J., Richardson A.L., Long L.J. (2022). Six month trajectories of COVID-19 experiences and associated stress, anxiety, depression, and impairment in American adults. Cogn. Ther. Res..

[109] Robinson E., Sutin A.R., Daly M., Jones A. (2022). A systematic review and meta-analysis of longitudinal cohort studies comparing mental health before versus during the COVID-19 pandemic in 2020. J. Affect. Disord..

[110] Palmas G., Moriondo M., Trapani S., Ricci S., Calistri E., Pisano L., Perferi G., Galli L., Venturini E., Indolfi G. (2020). Nasal swab as preferred clinical specimen for COVID-19 testing in children. Pediatr. Infect. Dis. J..

[111] Daly M., Robinson E. (2020). Psychological distress and adaptation to the COVID-19 crisis in the United States. J. Psychiatr. Res..

[112] Infurna F.J., Luthar S.S. (2018). Re-evaluating the notion that resilience is commonplace: A review and distillation of directions for future research, practice, and policy. Clin. Psychol. Rev..

[113] Robinson E., Daly M. (2021). Explaining the rise and fall of psychological distress during the COVID-19 crisis in the United States: Longitudinal evidence from the Understanding America study. Br. J. Health Psychol..

[114] Fancourt D., Steptoe A., Bu F. (2020). Trajectories of anxiety and depressive symptoms during enforced isolation due to COVID-19 in England: A longitudinal observational study. Lancet Psychiatry.

[115] Giebel C., Lord K., Cooper C., Shenton J., Cannon J., Pulford D., Shaw L., Gaughan A., Tetlow H., Gabbay M. (2021). A UK survey of COVID-19 related social support closures and their effects on older people, people with dementia, and carers. Int. J. Geriatr. Psychiatry.

[116] Wolitzky-Taylor K.B., Castriotta N., Lenze E.J., Stanley M.A., Craske M.G. (2010). Anxiety disorders in older adults: A comprehensive review. Depress. Anxiety.

[117] Hinz A., Brähler E. (2011). Normative values for the hospital anxiety and depression scale (HADS) in the general German population. J. Psychosom. Res..

[118] Girgus J.S., Yang K., Ferri C.V. (2017). The gender difference in depression: Are elderly women at greater risk for depression than elderly men?. Geriatrics.

[119] Pilania M., Yadav V., Bairwa M., Behera P., Gupta S.D., Khurana H., Mohan V., Baniya G., Poongothai S. (2019). Prevalence of depression among the elderly (60 years and above) population in India, 1997–2016: A systematic review and meta-analysis. BMC Public Health.

[120] Levkovich I., Shinan-Altman S., Essar Schvartz N., Alperin M. (2021). Depression and health-related quality of life among elderly patients during the COVID-19 pandemic in Israel: A cross-sectional study. J. Prim. Care Community Health.

[121] Byers A.L., Vittinghoff E., Lui L.Y., Hoang T., Blazer D.G., Covinsky K.E., Ensrud K.E., Cauley J.A., Hillier T.A., Fredman L. (2012). Twenty-year depressive trajectories among older women. Arch. Gen. Psychiatry.

[122] Sutin A.R., Terracciano A., Milaneschi Y., An Y., Ferrucci L., Zonderman A.B. (2013). The trajectory of depressive symptoms across the adult life span. JAMA Psychiatry.

[123] Borowiak E., Kostka T. (2004). Predictors of quality of life in older people living at home and in institutions. Aging Clin. Exp. Res..

[124] Salguero A., Martínez-García R., Molinero O., Márquez S. (2011). Physical activity, quality of life and symptoms of depression in community-dwelling and institutionalized older adults. Arch. Gerontol. Geriatr..

[125] Rodda J., Walker Z., Carter J. (2011). Depression in older adults. BMJ.

[126] Saraiva M.D., Apolinario D., Avelino-Silva T.J., de Assis Moura Tavares C., Gattás-Vernaglia I.F., Marques Fernandes C., Juliano M. (2021). The impact of frailty on the relationship between life-space mobility and quality of life in older adults during the COVID-19 pandemic. J. Nutr. Health Aging.

[127] El Haj M., Altintas E., Chapelet G., Kapogiannis D., Gallouj K. (2020). High depression and anxiety in people with Alzheimer’s disease living in retirement homes during the covid-19 crisis. Psychiatry Res..

